# Role of Prostate Apoptosis Response 4 in Translocation of GRP78 from the Endoplasmic Reticulum to the Cell Surface of Trophoblastic Cells

**DOI:** 10.1371/journal.pone.0080231

**Published:** 2013-11-25

**Authors:** Marie Cohen, Pascale Ribaux, Manuella Epiney, Olivier Irion

**Affiliations:** Department of Gynaecology Obstetrics, Faculty of Medicine, Switzerland; Institute of Molecular and Cell Biology, Biopolis, United States of America

## Abstract

Glucose-regulated protein 78 (GRP78) is an endoplasmic reticulum (ER) molecular chaperone that belongs to the heat shock protein 70 family. GRP78 is also present on the cell surface membrane of trophoblastic cells, where it is associated with invasive or fusion properties of these cells. Impaired mechanism of GRP78 relocation from ER to the cell surface was observed in preeclamptic cytotrophoblastic cells (CTB) and could take part in the pathogenesis of preeclampsia. In this study, we have investigated whether prostate apoptosis response 4 (Par-4), a protein identified as a partner of GRP78 relocation to the cell surface in prostate cancer cells, is present in trophoblastic cells and is involved in the translocation of GRP78 to the cell surface of CTB. Par-4 is indeed present in trophoblastic cells and its expression correlates with expression of membrane GRP78. Moreover, overexpression of Par-4 led to an increase of cell surface expression of GRP78 and decreased Par-4 gene expression reduced cell surface localization of GRP78 confirming a role of Par-4 in relocation of GRP78 from ER to the cell surface. Accordingly, invasive property was modified in these cells. In conclusion, we show that Par-4 is expressed in trophoblastic cells and is involved in transport of GRP78 to the cell surface and thus regulates invasive property of extravillous CTB.

## Introduction

GRP78 is an ER molecular chaperone that belongs to the heat shock protein 70 family (for a review [1]). The primary functions of GRP78 are related to its capacity to bind hydrophobic regions on nascent polypeptides in the ER and to its pivotal role in the signalling cascade producing the unfolded protein response (UPR) [2]. GRP78 expression can be stimulated by a variety of environmental and physiological stress conditions such as glucose starvation or hypoxia [3], [4]. GRP78 is well-known to reside inside the ER lumen. However, this chaperone is also located at the cell surface of cancer cells and cells undergoing ER stress [5] [4]. The mechanisms responsible for the translocation of this protein from the ER to the cell surface remain poorly understood [6]. The KDEL sequence of GRP78 present in its C-terminal part is involved in maintaining proteins within the ER lumen. It was thus hypothesized that overexpression of GRP78 observed under stress conditions may exceed the retention capacity of the KDEL retrieval system, resulting in relocation of GRP78 from the ER to the cell surface [7]. It was also hypothesized that the masking of the KDEL may be implicated in GRP78 transport to the cell surface. Additionally, particular GRP78-interacting protein partners are involved in the transport of GRP78 from the ER to the cell surface, and this can be cell-type-specific [6]. For example, MTJ-1 binds GRP78 and silencing MTJ-1 expression decreases cell-surface GRP78 expression in macrophages [8]. In prostate cancer cells, Par-4 seems to be required for the translocation of GRP78 from the ER to the plasma membrane [9]. On the outer plasma membrane, GRP78 functions as a receptor for a wide variety of ligands [2] and several small proteins can bind to surface GRP78 and modulate properties of cells [5].

Compared to normal tissue, tumours are subject to stress due to elevated glycolytic activity, inadequate blood vessel, creating a microenvironment of glucose deprivation, acidosis, and hypoxia [1]. Under such conditions, the level of GRP78 expression is highly induced and becomes essential for cell survival [1]. Its expression has been implicated in proliferation, invasion, apoptosis or cell survivaland drug resistance processes [10]–[16]. Indeed, knock down of GRP78 inhibits tumour cell invasion *in vitro* [17]. Similar observations were noted with tumour growth and metastasis in xenograft models [3], [7], suggesting an important role of GRP78 in cancer progression. However, the mechanism whereby GRP78 confers growth advantage to tumour cell is just emerging. The presence of GRP78 on the cell surface of metastatic cancer cells tends to suggest that it may mediate signal transduction pathways that induce proliferation and invasion [18].

Recently, we have demonstrated that GRP78 was highly expressed in trophoblastic cells and could also be found on these cells surface [19]. Trophoblasts are specialized cells of the placenta that are necessary for the formation of fetomaternal interface. Trophoblastic cells differentiate according to the villous or the extravillous pathway [20]. In the extravillous pathway, extravillous cytotrophoblastic cells (evCTBs) proliferate and differentiate into an invasive phenotype [20]. These cells invade decidual stromal compartments as well as spiral arteries of the decidua and the proximal third of the myometrium during the first trimester of pregnancy [21]. In the villous pathway, villous cytotrophoblastic cells (vCTBs) remain in the foetal compartment and fuse to form the syncytiotrophoblast (STB) [22]. STB is a multinuclear tissue forming the outer surface of the foetal part of the placenta and is crucial throughout pregnancy since it exerts unique specialized functions such as hormone secretion and generation of an immunological barrier [23], [24].

In primary first trimester CTBs (evCTB+vCTB), membrane GRP78 was shown to interact with and inactivate p53 [19]. This distribution pattern of GRP78 and p53 is surprising but reveals another common trait between CTBs and cancer cells [19]. In these cells, overexpression and localisation at the cell surface of GRP78 are also associated with *in vitro* invasive properties of trophoblastic cells as observed in various cancer cells [19], [25].

GRP78 autoantibodies and GRP78 proteins were found in the plasma of pregnant women. Interestingly, these autoantibodies and the ratio of C-terminal GRP78 products over total GRP78 were significantly lower in the plasma of first trimester pregnant women who will subsequently develop preeclampsia (PE) [25]. Development of PE is a two-stage process characterised by abnormal placentation, vascular remodelling and subsequent maternal syndrome marked by endothelial injury and activation. This disease is associated with or induced by defects in trophoblast invasion [23], confirming the potent role of GRP78 in the invasive properties of CTB. Moreover, whereas protein expression of GRP78 is not different in PE CTB compared to control CTB, expression of membrane GRP78 is significantly decreased in PE CTB suggesting a possible impaired mechanism of GRP78 relocation in PE CTB [26]. However, this mechanism remains unknown in trophoblastic cells.

Since mRNA of Par-4 was found in placenta [27], we propose to evaluate the role of Par-4 in transport of GRP78 from the ER to the cell surface of evCTBs and confirm the role of membrane GRP78 in trophoblastic cell invasion.

## Results

### Presence of Par-4 in Trophoblastic Cells

The presence of Par-4 in trophoblastic cells has never been reported. To test the hypothesis that Par-4 is involved in the transport of GRP78 from the ER to the cell surface of trophoblastic cells, we first evaluated the presence of Par-4 in these cells.

**Figure 1:**
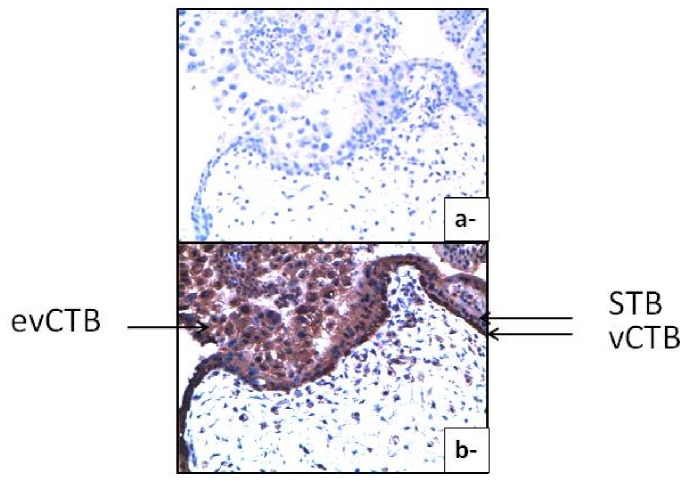
Expression of Par-4 in trophoblastic cells. Immunohistochemistry of first trimester trophoblast with control IgG (a) or Par-4 antibodies (b). Magnification: x40.

As shown in figure 1, Par-4 is observed in extravillous (ev) and villous (v) cytotrophoblast (CTB) and syncytiotrophoblast (STB). It is mainly immunolocalised in the cytoplasm of STB and evCTB but is also strongly stained in both nucleus and cytoplasm of vCTB.

**Figure 2:**
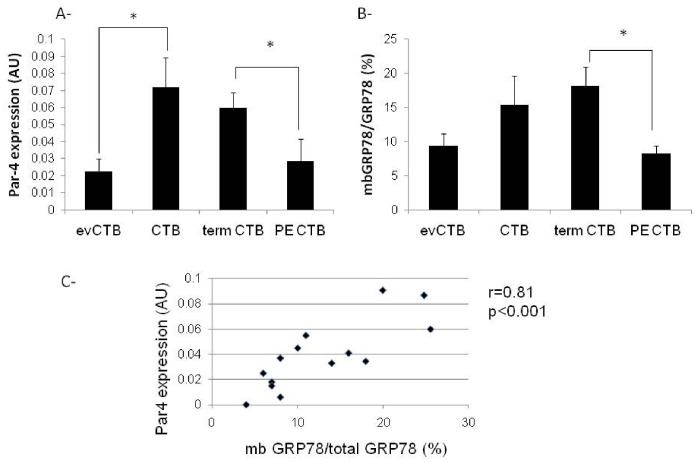
Correlation of Par-4 and GRP78 expression in trophoblastic cells. A- Cell-ELISA of Par-4. on cytotrophoblastic cells (CTB). eV = extravillous, PE = preeclamptic B- Cell-ELISA of GRP78 on cytotrophoblastic cells (CTB). eV = extravillous, PE = preeclamptic. Data are expressed as the ratio of membrane (mb) over total GRP78 in percent. C- Correlation curve between Par-4 and membrane GRP78 expression.

We then evaluated the expression of total Par-4 and membrane GRP78 in purified first trimester CTB, evCTB, term vCTB and PE vCTB by cell-ELISA (figure 2A and B). Expression of Par-4 and membrane GRP78 is significantly lower in first trimester evCTB than in CTB (first trimester or term CTB). Moreover, the level of Par-4 and membrane GRP78 is significantly decreased in PE vCTB compared to control vCTB (term CTB). We also found a significant correlation between Par-4 and membrane GRP78 expression of these cells (figure 2C).

### Role of Par-4 in the Transport of GRP78 from the ER to the Cell Surface

In the next series of experiments, we evaluated the role of Par-4 in the cell surface translocation of GRP78 by overexpressing or silencing Par-4 using an expression plasmid or a siRNA respectively. We proposed that if Par-4 is indeed involved in the translocation of GRP78 to the cell surface, then overexpression or silencing of the Par-4 gene would respectively increase or limit the availability of Par-4 protein for translocation of GRP78 to the cell surface, causing a change in the level of cell surface GRP78.

We have first investigated the effects of exogenous Par-4 on GRP78 expression and localisation of an extravillous trophoblastic cell line (HIPEC 65) which is easier to transfect than primary evCTB. Membrane expression of GRP78 was evaluated by Cell-ELISA and western blot analysis of membrane proteins. GAPDH was used as control of loading for cytosolic proteins and as purity control of membrane fraction. Actin was used as control of loading for both cytosolic and membrane proteins.

**Figure 3:**
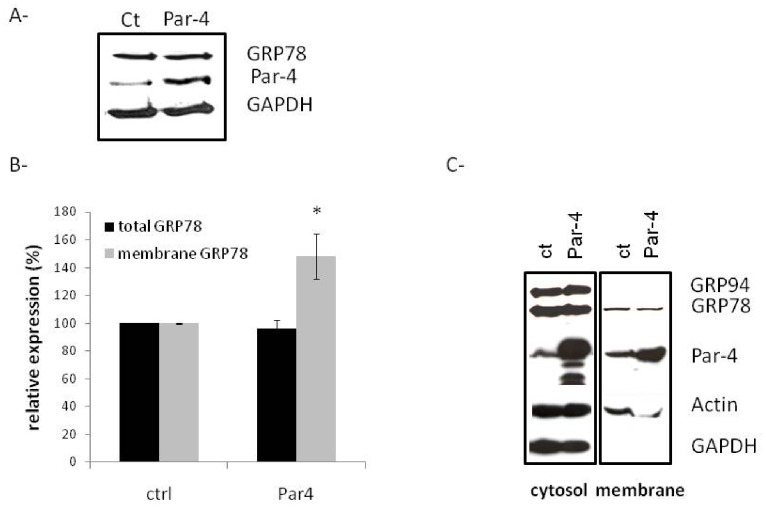
Effect of Par-4 overexpression on GRP78 localization in HIPEC 65 cells. Cells were transfected with control (Ctrl) or Par-4 expressing plasmid and incubated for 48 h before harvesting. A- Western blot of protein extracts was performed under reducing conditions and probed with anti-GRP78, Par-4 or GAPDH antibodies. B- Cell-ELISA of total and membrane (mb) GRP78. *p<0.05. C- Western blot of cytosolic and membrane proteins was performed under reducing conditions and probed with anti-GRP78, Par-4, Actin and GAPDH antibodies.

Overexpression of Par-4 did not influence total GRP78 protein expression (figure 3A,), but increased significantly the relocation of GRP78 at the cell surface of HIPEC 65 cellsevaluated by Cell-ELISA (figure 3B) or western blot (figure 3C). Because overexpression of Par-4 did not affect GRP78 expression, but affected its cell surface expression, it was suggested that Par-4 availability affects the cell surface expression of GRP78.

**Figure 4:**
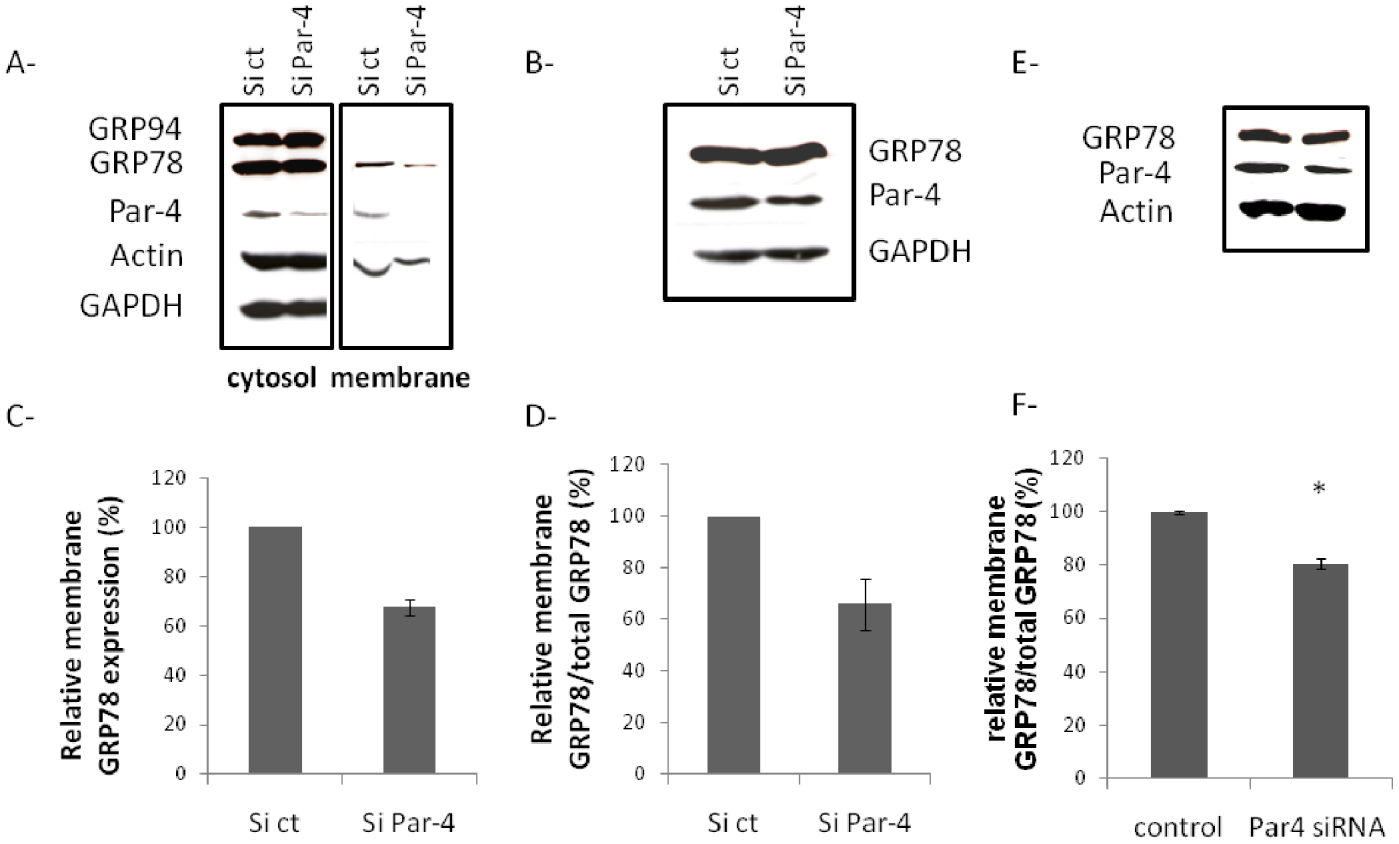
Effect of Par-4 reduction on GRP78 localisation in evCTB. HIPEC 65 cells (A- to D-) or primary evCTB (E-, F-) were transfected with control (ctrl) or Par-4 siRNA for 24 h. Cells were then trypsinized, plated and cultured for 48 h before protein extraction and Cell-ELISA, or incubated for 72 h for subcellular fractionation. A- Western blot of cytosolic and membrane proteins was performed under reducing conditions and probed with anti-GRP78, Par-4, Actin and GAPDH antibodies. B- Western blot of protein extracts (40 µg) was performed under reducing conditions and probed with anti-GRP78, Par-4 or GAPDH antibodies. C- Quantification of membrane GRP78 expression from 3 independent experiments of western blot. D- Cell-ELISA of GRP78 on HIPEC 65 cells. Data are expressed as the relative ratio of membrane (mb) over total GRP78 in percent. *p<0.05. E- Western blot of protein extracts (40 µg) from primary evCTB was performed under reducing conditions and probed with anti-GRP78, Par-4 or GAPDH antibodies. F- Cell-ELISA of GRP78 on primary evCTB. Data are expressed as the relative ratio of membrane (mb) over total GRP78 in percent. *p<0.05.

To confirm this hypothesis, we next evaluated the role of endogenous Par-4 in GRP78 expression and localisation in HIPEC 65 cells. Transfection of these cells with Par-4 siRNA led to a significant decrease of both Par-4 expression (figure 4A and B) and membrane GRP78 expression evaluated by western blot (figure 4A, B or Cell-ELISA (figure 4D). We next examined the role of endogenous Par-4 in membrane GRP78 expression in primary evCTB. As shown in figure 4, decreased expression of Par-4 (figure 4E) led to a slight but significant decrease in GRP78 membrane expression (figure 4F).

### Regulation of Invasive Properties in Transfected Trophoblastic Cells

**Figure 5:**
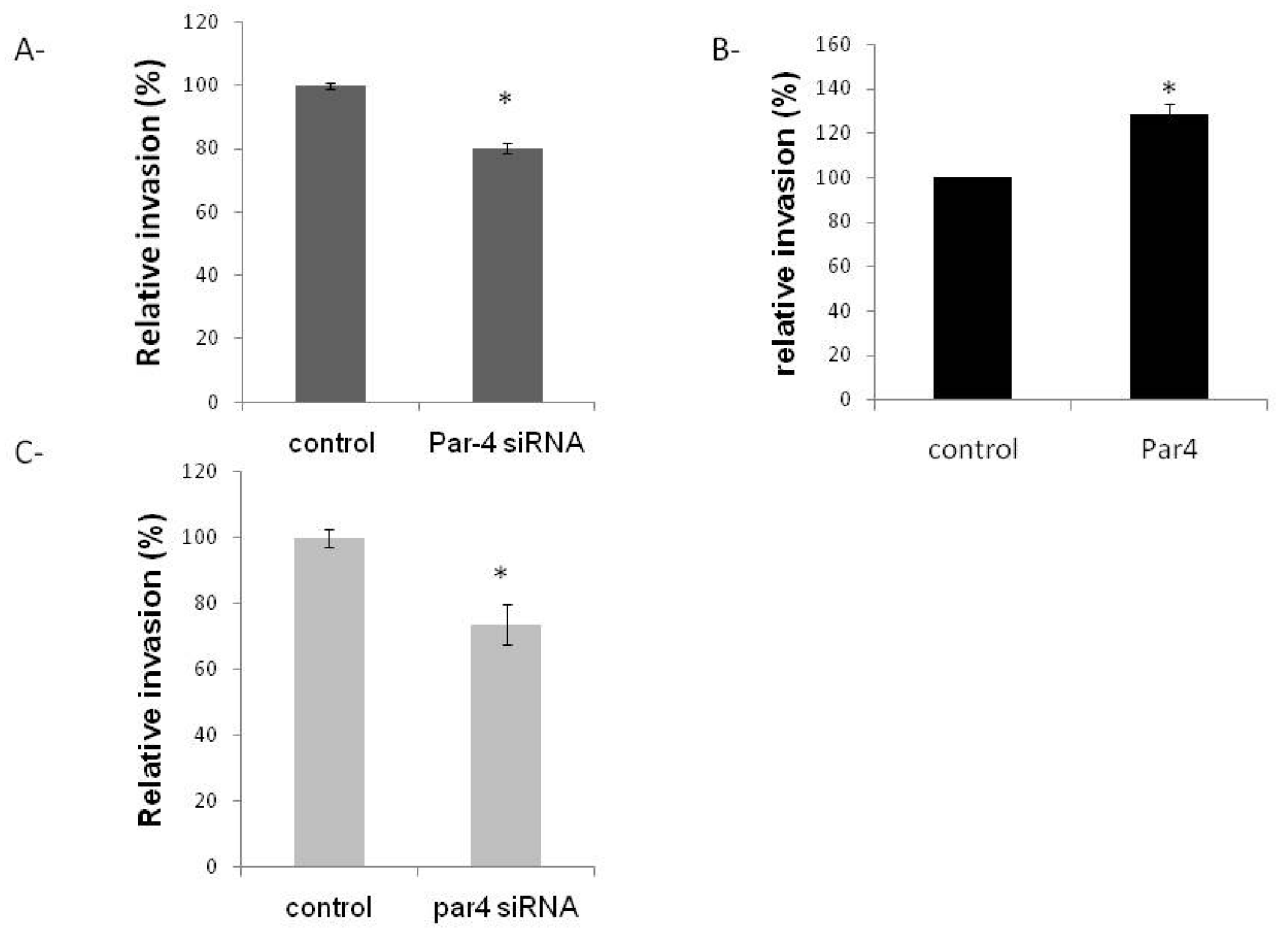
Effect of Par-4 on invasive property of trophoblastic cells. HIPEC 65 cells were transfected with control or Par-4 siRNA (A-) or control (ctrl) or Par-4 expressing plasmid (B-) for 24 h. Cells were then trypsinized and seeded on collagen I precoated transwell membranes for 48 h. Cells that invaded collagen were quantified by colorimetric assay. Data were expressed as the percentage of transfected cells that invaded the collagen-coated membrane relative to the control cells. C- Invasive properties of primary evCTB transfected with control (ctrl) or Par-4 siRNA. *p<0.05.

We have previously reported that membrane GRP78 is involved in invasive properties of evCTB [19], [25]. To further confirm the role of Par-4 in the relocation of GRP78 at the cell surface, we have evaluated the invasive property of HIPEC 65 cells transfected with Par-4 expressing plasmid or siRNA. Silencing of Par-4 gene expression led to a decrease of HIPEC 65 cell invasion (figure 5A). In contrast, overexpression of Par-4 led to an increase of HIPEC 65 cell invasion (figure 5B). To validate the hypothesis that Par-4 plays an important role in regulating evCTB invasion, primary evCTB were also transfected with Par-4 siRNA. As observed in figure 5C, decreased expression of Par-4 led to a slight but significant decrease in evCTB invasion. These effects observed in both primary and evCTB cell line are independent of cell viability since we confirmed that expression of Par-4 did not influence cell viability (data not shown).

## Discussion

Par-4 is a multi-domain protein of 340 amino acids. The key domains are highly conserved among different species and are leucine zipper domain at the C-terminal part, two nuclear localization sequences at the N-terminal part, a nuclear export sequence and the SAC (Selective for Apoptosis of Cancer cells) domain. In addition, Par-4 contains a number of conserved consensus sites for phosphorylation by kinases suggesting that Par-4 may be tightly regulated by post-translational modification, localization and interactions with proteins of biological consequence. The main biological role of Par-4 described in literature is pro-apoptotic and depends on interactions with other proteins such as PKCζ, WT-1, ZIP kinase, THAP-1 [9], [28], [29]. More generally, functions of Par-4 are dependent on its partners. Secreted Par-4 could also induce apoptosis and this would be rendered possible by its interaction with cell surface GRP78 in cancer cells [9]. In this last study, Par-4 knock-down does not alter total GRP78 levels in whole-cell lysates, but decreases the cell surface GRP78 expression in prostate cancer cells, suggesting that endogenous Par-4 is involved in the cell surface expression of GRP78.

Here, we have reported for the first time the presence of Par-4 in both villous and extravillous trophoblastic cells. Its expression in these cells is significantly correlated with the presence of membrane GRP78 but not with total GRP78 expression, suggesting a role for Par-4 in the GRP78 transport from the ER to the cell surface in trophoblastic cells.

Upregulation or downregulation of Par-4 expression in HIPEC 65 cells and evCTB respectively leads to a change in membrane expression of GRP78, confirming the hypothesis that Par-4 plays a role in the transport of GRP78 from the ER to the cell surface in trophoblastic cells. Consistent with previous works suggesting that membrane GRP78 is involved in the regulation of evCTB invasion [19], [25], downregulation or upregulation of cell surface GRP78 induced by down regulation or upregulation of Par-4 respectively, influences the invasive property of evCTB. Thus, Par-4 is involved in the transport of GRP78 from the ER to the cell surface of evCTB and therefore regulates indirectly trophoblastic cell invasion.

By immunohistochemistry, we have observed the presence of Par-4 in villous CTB and syncytiotrophoblast, notoriously non-invasive trophoblasts. Moreover, we have recently shown that membrane GRP78 is involved in the trophoblastic differentiation of villous CTB into syncytiotrophoblast [26], suggesting that Par-4 may also be indirectly implicated in trophoblastic differentiation.

In a previous study, we have observed that GRP78 protein level is not modified in preeclamptic (PE) vCTB compared to control vCTB. In contrast, membrane GRP78 level is decreased in PE vCTB compared to control ones [26]. Since GRP78 protein level is not different between PE vCTB and control vCTB, the decreased membrane GRP78 expression observed in PE vCTB is probably due to an altered GRP78 translocation from the ER to the plasma membrane. Therefore, the decreased expression of the cell surface GRP78 in PE vCTB could be due to an altered expression of GRP78 transporting proteins. Interestingly, we have found that Par-4 protein expression is decreased in PE CTB compared to control ones. Nevertheless, expression of Par-4 mRNA was not significantly different between these two types of cells (not shown). In this context, it would be interesting to study the expression, localisation, post-translational modifications and half-life of Par-4 in these cells to better understand the dysfunctions responsible for PE.

## Materials and Methods

### Ethics Statement

This research has been approved by the departmental ethics committee of maternity and pediatrics, University Hospital of Geneva (10-001 and 02-088). Informed written consent was obtained from all patients before their inclusion in the study.

### Purification of Primary Trophoblastic Cells

Placental tissue was obtained from patients undergoing a legal abortion during the first trimester (8–12 weeks of gestation, n = 4 for purification of evCTB and n = 3 for purification of CTB) or at delivery from normal (n = 3) or preeclamptic patients (n = 4). CTB were isolated as previously described [30]. In brief, fresh tissue specimens were isolated and washed several times in sterile Hanks balanced salt solution (HBSS). Tissue was then enzymatically digested five times for 20 min at 37°C (0.25% trypsin, 0.25 mg/ml Dnase I; Roche, Diagnostics GmbH, USA). After incubation, the trypsin cocktail was neutralized with fetal bovine serum (FBS), and the cells resuspended in Dulbecco’s modified Eagle’s medium (DMEM) (Invitrogen, Switzerland). This cell suspension was filtered through a 50-μm mesh, laid onto a Percoll gradient (70-5% Percoll diluted with HBSS) and centrifuged for 25 min at 1200 g. The 30–45% percoll layer containing trophoblast cells was collected, the cells washed and resuspended in DMEM. The cells were then immunopurified with immobilized anti-CD45 antibodies. Ninety five percent of cells were positive for cytokeratin 7 and negative for vimentin.

To obtain primary evCTB, the immunopurified cells were seeded on Petri dishes for 15 min. Supernatant containing evCTB was centrifuged and the cells were resuspended in culture medium. Ninety five percent of 24 h cultured evCTB were positive for cytokeratin 7 and HLA-G and negative for vimentin.

### Cell Culture

HIPEC 65 cells were generously given by Thierry Fournier (Inserm U767, Paris, France). HIPEC 65 cells and primary evCTB were grown in DMEM supplemented with 10% (v/v) FBS and gentamicin under 5% CO2, at 37°C.

### Transfection

To study the effect of Par-4 overexpression on GRP78 localisation, 1 million of HIPEC 65 cells were plated in 100 mm dishes and then transfected with Par-4 expressing plasmid (gift from Professor Rangnekar, University of Kentucky, Lexington, USA) or control plasmid (25 nM). JetPei transfection reagent was used to transfect cells as described by the manufacturer (Polyplus transfection, Illkirch, France). After 24 h, cells were trypsinized and seeded in insert (for invasion assay), 96 wells (for MTT assay and Cell-ELISA) and 3 cm dishes (for protein analysis) for 48 h.

To study the effect of endogenous Par-4 on GRP78 localisation in HIPEC 65 cells, 3 millions or 1 million of cells were plated in 100 mm or 60 mm respectively dishes and then transfected with Par-4 or control siRNA using the Interferin tranfection reagent (Polyplus transfection Illkrich, France) and following manufacturer’s protocol. After 24 h, cells plated in 60 mm dishes were trypsinized and seeded in inserts (for invasion assay), 96 wells (for MTT assay and Cell-ELISA) and 3 cm dishes (for protein analysis) for 48 h. Cells plated in 100 mm dishes were incubated 72 h before subcellular fractionation.

To study the effect of endogenous Par-4 on GRP78 localisation of primary evCTB, 20 millions of evCTB were plated in 100 mm dishes and then transfected with Par-4 siRNA or control siRNA (Santa Cruz Biotechnology, Labforce, Switzerland, 25 nM). Lipofectamine transfection reagent was used to transfect cells as described by the manufacturer. After 24 h, cells were trypsinized and seeded in inserts (for invasion assay), 96 wells (for MTT assay and Cell-ELISA) and 3 cm dishes (for protein analysis) for 48 h.

Each experiment was performed three times with different cell preparations and run in triplicate.

### Immunohistochemistry

Placental tissues obtained from first trimester legal abortions were rapidly washed with 0.1 mol/l phosphate buffered saline (PBS) at pH 7.4 and fixed for 4–12 h in 4% buffered formalin at 4°C. The specimens were then dehydrated in ethanol and embedded in paraffin wax.

Serial sections of tissue were deparaffinized and rehydrated through graded ethanol. Antigen retrieval was performed by microwave pretreatment in 0.01 mol/L citrate buffer (pH 6.0) for 5 minutes four times, followed by cooling in a cold water bath. Non-specific binding was blocked with 3% (v/v) bovine serum albumin (BSA) in PBS for 30 minutes at room temperature. The sections were incubated with a primary antibody specific for Par-4 (R-334, Santa Cruz Biotechnology, Labforce, Switzerland, dilution 1/20 in 3% BSA-PBS) overnight at 4°C. Control sections were incubated with control rabbit IgG in 3% BSA-PBS overnight at 4°C. Sections were then washed with PBS and incubated with horse radish peroxydase (HRP) conjugated anti-mouse secondary antibodies (dilution 1/500) for 1 h. After washing, trophoblast cuts were stained with diaminobenzidine (DAB) chromogen system (Dako, Baar, Switzerland). Nuclei were counterstained with hemalun (Sigma-Aldrich, Saint Louis, USA).

Evaluation of the different trophoblastic cell types was based on their morphology and was confirmed by two placental specialists (Dr Paul Bischof and Dre Vildana Finci, Geneva, Switzerland).

### Cell ELISA

Primary trophoblastic cells or HIPEC 65 cells were seeded at 100 000 cells/well or 10 000 cells/well respectively in a 96-well plate and incubated for 48 h. Cells were either incubated directly with the primary antibodies (for detection of membrane proteins) or washed, fixed (3% paraformaldehyde in PBS), permeabilized (0.2% triton in PBS), pre-incubated with 3% BSA-PBS (30 minutes) (for detection of both intracellular and membrane proteins) before incubation with the primary antibodies. The following antibodies: anti-GRP78 antibodies (Gl-19, Sigma-Aldrich, Saint Louis, USA, dilution 1/500),or anti-Par-4 antibodies (A-10, Santa Cruz Biotechnology, Labforce, Switzerland, dilution 1/100) were incubated with the cells for 45 minutes at 4°C. To remove the unbound antibody, cells were washed four times in PBS-BSA3%, and then incubated 30 minutes at 4°C with horseradish peroxidase (HRP) conjugated goat anti-rabbit or anti-mouse IgG antibody (Santa Cruz Biotechnology, Labforce, Switzerland, dilution 1/500). After incubation, cells were washed as described above and the substrate 3,3′,5,5′-tetramethyl benzidine (R&D systems, Minneapolis, USA) was added. The reaction was stopped by adding 1 M sulfuric acid. Absorbance was read at 450 nm on a microplate reader (Expert plus, Biochrom). This experiment was performed three times with different cell preparations and run in triplicates.

### Subcellular Fractionation

Subcellular fractionation was realised using membrane protein extraction kit (Promokine, Heudeberg, Germany) according to the manufacturer’s protocol.

### Western Blot

Whole cell extracts (40 µg of proteins) or subcellular fractions were fractionated by SDS-Page 10% and transferred to nitrocellulose membrane for immunoblot analysis using rabbit anti-GRP78 antibodies (1∶3000, GL-19, Sigma-Aldrich), mouse anti-Par-4 antibodies (3G9H7, Santa Cruz Biotechnology, Labforce, Switzerland, dilution 1/3000), mouse anti-actin antibodies (dilution 1∶3000 from SantaCruz Biotechnology) and mouse anti-GAPDH antibodies (1∶30 000 dilution from Millipore, Temecula, CA, USA).

### Invasion Assay

Cell invasion assay was performed in an invasion chamber as described elsewhere [19]. Briefly, 1×10^4^ HIPEC 65 cells or 1×10^5^ evCTB in 100 µl of medium were added to the upper compartment of the transwell chambers. Medium (400 µl) was added in the lower chamber. After 48 h of incubation at 37°C, viable cells that invaded collagen were stained with crystal violet and colorimetric measurement was performed at 560 nm. This assay was repeated three times and each experiment was run in triplicate. Data were expressed as the percentage of treated cells that invaded the collagen-coated membrane compared to the untreated (controls) cells.

### Proliferation/Viability Assay

To determine the effect of Par4 expression on cell viability and evaluate the seeding of cells for invasion assay, we performed a MTT assay. 3×10^4^ HIPEC 65 cells or 1×10^5^ evCTB were seeded in 96-well plates and incubated in 100 µl of medium for 48 h before being replaced with medium containing 20% MTT (Sigma-Aldrich Corporation, USA) solution (5 mg/ml in medium) for 2 h. Acidic isopropanol solution (150 µl) was added, and then each well was vigorously mixed to dissolve the precipitated formazan. UV–visible absorption was measured at 560 and 690 nm.

### qRT-PCR

Reverse transcription was performed with 1 µg of total RNA in a final volume of 20 µl using High Capacity cDNA Reverse Transcription Kit (Applied biosystems, Foster city, USA). The quantitative detection of the PCR product was performed using the KAPA SYBR FAST Universal qPCR Kit (KAPA Biosystems, Boston, USA), with the iCycler iQ System (Bio-Rad).

The relative expression of Par-4 and GRP78 mRNA was normalized to the housekeeping genes GAPDH, HPRT1 and cyclophilin A.

Oligonucleotide primers for qPCR are listed in table 1.

**Table 1:** Sequence of primers used for qPCR.

GAPDH	forward 5′-CGACCACTTTGTCAAGCTCA-3′reverse 5′-CCCTGTTGCTGTAGCCAAAT-3′
HPRT1	forward 5′-ATGACCAGTCAACAGGGGAC-3′reverse 5-TGCCTGACCAAGGAAAGCAA-3′
Cyclophilin A	forward 5′-GTTTGCAGACAAGGTCCCA-3′reverse 5′-ACCCGTATGCTTTAGGATG-3′
Par-4	forward 5′-CTGCCGCAGAGTGCTTAGAT-3′reverse 5′-CATCTTCTCGTTTCCGCTCT-3′
GRP78	forward 5′-CGTGGAGATCATCGCCAAC-3′reverse 5′-AGCAATCTGGGCCTCAAAGAT-3

### Statistical Analysis

Results were expressed as mean +/− standard deviation. The difference between samples was evaluated by the Student’s t test with p<0.05 considered as statistically significant.
